# *In vivo* screening for toxicity-modulating drug interactions identifies antagonism that protects against ototoxicity in zebrafish

**DOI:** 10.3389/fphar.2024.1363545

**Published:** 2024-03-07

**Authors:** Ethan Bustad, Emma Mudrock, Elizabeth M. Nilles, Andrea Mcquate, Monica Bergado, Alden Gu, Louie Galitan, Natalie Gleason, Henry C. Ou, David W. Raible, Rafael E. Hernandez, Shuyi Ma

**Affiliations:** ^1^ Center for Global Infectious Disease Research, Seattle Children’s Research Institute, Seattle, WA, United States; ^2^ Department of Otolaryngology-HNS, University of Washington, Seattle, WA, United States; ^3^ Department of Biological Structure, University of Washington, Seattle, WA, United States; ^4^ Department of Biology, University of New Mexico, Albuquerque, NM, United States; ^5^ Department of Pediatrics, Seattle Children’s Hospital, Seattle, WA, United States; ^6^ VM Bloedel Hearing Research Center, University of Washington, Seattle, WA, United States; ^7^ Department of Pediatrics, University of Washington, Seattle, WA, United States; ^8^ Department of Chemical Engineering, University of Washington, Seattle, WA, United States; ^9^ Pathobiology Graduate Program, Department of Global Health, University of Washington, Seattle, WA, United States

**Keywords:** ototoxicity, otoprotection, toxicity protection, drug-drug interactions, zebrafish, aminoglycosides, macrolides

## Abstract

**Introduction:** Ototoxicity is a debilitating side effect of over 150 medications with diverse mechanisms of action, many of which could be taken concurrently to treat multiple conditions. Approaches for preclinical evaluation of drug-drug interactions that might impact ototoxicity would facilitate design of safer multi-drug regimens and mitigate unsafe polypharmacy by flagging combinations that potentially cause adverse interactions for monitoring. They may also identify protective agents that antagonize ototoxic injury.

**Methods:** To address this need, we have developed a novel workflow that we call Parallelized Evaluation of Protection and Injury for Toxicity Assessment (PEPITA), which empowers high-throughput, semi-automated quantification of ototoxicity and otoprotection in zebrafish larvae via microscopy. We used PEPITA and confocal microscopy to characterize in vivo the consequences of drug-drug interactions on ototoxic drug uptake and cellular damage of zebrafish lateral line hair cells.

**Results and discussion:** By applying PEPITA to measure ototoxic drug interaction outcomes, we discovered antagonistic interactions between macrolide and aminoglycoside antibiotics that confer protection against aminoglycoside-induced damage to lateral line hair cells in zebrafish larvae. Co-administration of either azithromycin or erythromycin in zebrafish protected against damage from a broad panel of aminoglycosides, at least in part via inhibiting drug uptake into hair cells via a mechanism independent from hair cell mechanotransduction. Conversely, combining macrolides with aminoglycosides in bacterial inhibition assays does not show antagonism of antimicrobial efficacy. The proof-of-concept otoprotective antagonism suggests that combinatorial interventions can potentially be developed to protect against other forms of toxicity without hindering on-target drug efficacy.

## 1 Introduction

Ototoxicity is a debilitating side effect of over 150 medications used to treat a broad range of conditions, including cancer and recalcitrant infections (Lanvers-Kaminsky et al., 2017). The sensorineural hearing or balance impairments that result from ototoxicity harm patient quality of life and incurs follow-up costs averaging $300,000-$1 million per patient (Lanvers-Kaminsky et al., 2017). Adverse drug-drug interactions (DDIs) with the potential to exacerbate ototoxicity complicate the implementation of treatment for multiple concurrent conditions. Currently, many DDIs are discovered only after the drugs have reached market (Percha and Altman, 2013) as drug effects on the ear are not routinely tested in pre-clinical and clinical trials (Verdel et al., 2008). This late-stage discovery exacerbates the toll on patient health and financial costs. New approaches for preclinical identification of potential DDIs would mitigate unsafe polypharmacy by flagging regimens may increase toxicity for monitoring and even potentially identify protective agents that antagonize ototoxic injury.

Zebrafish (*Danio rerio*) are a well-established model organism for studying ototoxicity that offer the advantage of conserved vertebrate physiology, as well as compatibility with high-throughput assays typically limited to cell-based models (Hill et al., 2005; McGrath and Li, 2008; Raldua and Pina, 2014; Driessen et al., 2015; MacRae and Peterson, 2015; Rennekamp and Peterson, 2015; Rider et al., 2015). Zebrafish share a high degree of genetic similarity with humans (70% of human genes have a clear zebrafish ortholog (Howe et al., 2013)), and the zebrafish lateral line hair cells (HCs), which detect vibrations in water, are structurally and functionally homologous to HCs of the human inner ear that sense vibrations of sound waves to enable hearing (Chiu et al., 2008; Ou et al., 2010). Zebrafish larval lateral line HCs have functionality that more closely resembles that of mammalian HCs *in vivo* relative to cell lines derived from cochlear tissues, whilst also offering the potential for higher-throughput profiling experiments relative to *ex vivo* mammalian inner ear explant models that are laborious to establish (Barrallo-Gimeno and Llorens, 2022)*.* Importantly, studies have shown that zebrafish larval lateral line HCs are sensitive to the same drugs that cause ototoxicity in humans (Chiu et al., 2008; Ou et al., 2010), and that the majority of drug interactions tested in zebrafish have replicated in humans (MacRae and Peterson, 2015).

The gold standard assay for ototoxic drug screening in zebrafish requires expert evaluation of multiple, individual neuromasts; attempts have been made to automate the analysis process (Ton and Parng, 2005; Philip et al., 2018), but throughput remains limited, preventing systematic evaluation of DDI. To address this challenge, we have developed a novel workflow that we call Parallelized Evaluation of Protection and Injury for Toxicity Assessment (PEPITA) for high-throughput, semi-automated quantification of ototoxicity and otoprotection in zebrafish larvae. By combining robotics-assisted 96-well plate-based microscopy imaging of whole zebrafish larvae and computational image analysis of the lateral line, our workflow empowers quantification of HC damage in hundreds of fish per day. Besides enabling larger-scale drug screening for putative ototoxic and otoprotective agents, PEPITA also enables the quantification of DDI outcomes of combinatorial drug co-administration.

By applying PEPITA to characterize ototoxic drug-drug interaction outcomes, we have discovered an antagonistic interaction between macrolide and aminoglycoside antibiotics that confers protection against aminoglycoside-induced damage to lateral line HCs in zebrafish larvae. Co-administration of either azithromycin or erythromycin in zebrafish protected against damage from a broad panel of aminoglycosides. Interestingly, co-administration of these macrolides with aminoglycosides in bacterial growth assays do not show a corresponding antagonism of antimicrobial efficacy. The proof-of-concept otoprotective antagonism between macrolides and aminoglycosides suggest that combinatorial interventions can be developed that protect against other forms of toxicity without hindering on-target efficacy. The platform PEPITA empowers the systematic screening for candidate combinations that elicit these protective interactions in the context of ototoxicity.

## 2 Materials and methods

### 2.1 Zebrafish husbandry

All experiments involving live zebrafish were carried out in compliance with Seattle Children’s Research Institute’s (IACUC protocol number ACUC00658) and University of Washington’s Institutional Animal Care and Use Committee guidelines (IACUC protocol number 2997-01). As zebrafish lateral line HCs develop by 5 days post-fertilization (dpf), experiments were conducted when fish were 5 dpf unless otherwise noted. Sex is not determined at this age.

For drug dose response testing, zebrafish of the wildtype genetic background AB (Table 1) and fish that were transgenic for GFP under the *myo6b* HC-specific promoter (Tg (*myo6b:EGFP*), Table 1) were raised as described previously (Westerfield, 2007; Farr et al., 2020). Briefly, zebrafish embryos were collected from 2 h spawning periods, washed with dilute bleach solution (0.005% sodium hypochlorite) by 6 hours post-fertilization to minimize contamination from microbes, and raised in Petri dishes in ICS water (300 mg Instant Ocean/L, 0.56 mM CaCl_2_, 1.2 mM NaHCO_3_, Table 1) (Linbo et al., 2009) at a density of 50 larvae per 100-mm^2^ dish, in a dark 28.5°C incubator until 5 dpf, with fluorophore screening performed when relevant alongside dechorionation at 2–4 dpf; fish homozygous and heterozygous for fluorophore expression were both used.

**TABLE 1:** Key resources table.

Reagent type (species) or resource	Designation	Source or reference	Identifiers
Strain, zebrafish (*Danio rerio*)	AB	ZIRC	http://zebrafish.org/fish/lineAll.php?OID=ZDB-GENO-960809-7
Strain, bacteria	*Escherichia coli* ATCC 25922	ATCC	https://www.atcc.org/products/25922
Strain, bacteria	*Mycobacterium* abscessus ATCC 19977	ATCC	https://www.atcc.org/products/19977
Strain, bacteria	*Staphylococcus aureus* ATCC 29213	ATCC	https://www.atcc.org/products/29213
Genetic reagent (*Danio rerio*)	Tg (*myo6b:EGFP*)^w119Tg^; *myo6b::gfp*	Hailey et al. (2017)	https://zfin.org/ZDB-ALT-170321-13
Genetic reagent (*Danio rerio*)	Tg (*myo6b:mitoGCaMP3*)^w119Tg^; *mitoGCaMP3*	Esterberg et al. (2016)	https://zfin.org/ZDB-ALT-141008-1
Genetic reagent (*Danio rerio*)	Tg (*myo6b:RGECO1*); *cytoRGECO*	Maeda et al. (2014)	https://zfin.org/ZDB-ALT-150114-2
Chemical compound, drug	Amikacin sulfate	Sigma-Aldrich	PHR 1860-1G
Chemical compound, drug	Aspirin	ThermoFisher	18–600-802
Chemical compound, drug	Azithromycin dihydrate	ThermoFisher	AAJ6674006
Chemical compound, drug	Benzamil	Sigma-Aldrich	B2417-50 MG
Chemical compound, drug	Capreomycin sulfate	Sigma-Aldrich	PHR1716-500 MG
Chemical compound, drug	Cisplatin	Biotechne	2251
Chemical compound, drug	Clarithromycin	TCI	C2220
Chemical compound, drug	Cyclodextrin	ThermoFisher	AAH3113306
Chemical compound, drug	Cyclosporine A	ThermoFisher	AAJ6319106
Chemical compound, drug	Dexamethasone	ThermoFisher	AAA1759003
Chemical compound, drug	Dihydrostreptomycin	ThermoFisher	ICN19452805
Chemical compound, drug	Ebselen	ThermoFisher	AAJ63190MA
Chemical compound, drug	Erythromycin	ThermoFisher	BP920-25
Chemical compound, drug	Gentamicin sulfate	ThermoFisher	BP918-1
Chemical compound, drug	Kanamycin sulfate	ThermoFisher	BP9065
Chemical compound, drug	Mefloquine	ThermoFisher	AC461130010
Chemical compound, drug	Neomycin sulfate hydrate	ThermoFisher	AAJ6149914
Chemical compound, drug	Tobramycin	Sigma-Aldrich	T4014-500 MG
Chemical compound, drug	3-aminobenzoic acid ethyl ester, methanesulfonate salt (tricaine)	Pentair	TRS5
Chemical compound, fluorophore	Texas Red-X succinimidyl ester	ThermoFisher	T20175
Chemical compound, stain	YO-PRO-1 Iodide	ThermoFisher	Y3603
Media	ICS water; Instant Ocean Sea Salt	Spectrum Brands Product SS15-10	https://www.chewy.com/instant-ocean-sea-salt-aquariums/dp/196228
Media	Middlebrook 7H9 Broth	ThermoFisher	DF0713-17-9
Media	Cation-adjusted Mueller-Hinton Broth (CAMHB) with TES	ThermoFisher	T3462
Hardware	Non-treated 12 and 24 well plates	ThermoFisher	07–201-589, 07–201-590
Hardware	Non-treated 96 well clear, round bottom plates	GenClone	25–224
Hardware	Non-treated 96 well black, flat bottom imaging plates	VWR	37,000–550
Hardware	3D printer filament: PLA	Amazon	B084XS2TS9
Hardware	125 micron Nylon mesh	ELKO Filtering Co.	03–125/39
Software	Tinkercad design for netwells		https://www.thingiverse.com/thing:5676024
Software	ImageJ	National Institutes of Health	https://imagej.nih.gov/ij/download.html

For calcium testing, zebrafish expressing HC-specific GCaMP targeted to the inner mitochondrial matrix were crossed with fish expressing a HC specific calcium indicator to create double-transgenic embryos (Tg (*myo6b:mitoGCaMP3;myo6b:cytoRGECO*), Table 1) with both green fluorescent mitochondrial calcium and red fluorescent cytosolic calcium indicators, then raised as described previously (McQuate et al., 2023). Zebrafish embryos were collected from 2-h spawning periods and raised in Petri dishes in embryo medium (EM: 14.97 mM NaCl, 500 μM KCl, 42 µM Na_2_HPO_4_, 150 µM KH_2_PO_4_, 1 mM CaCl_2_ dehydrate, 1 mM MgSO_4_, 0.714 mM NaHCO_3_, pH 7.2) at a density of 60 larvae per 100-mm^2^ dish, in a dark 28.5°C incubator until 5 dpf. Larvae were screened for indicator expression at 4 dpf.

### 2.2 Drug response testing

Healthy zebrafish lateral line HCs are selectively permeable to fluorescent vital dyes including YO-PRO1 (Table 1), which selectively stains lateral HC nuclei (Ou et al., 2010). The extent of drug-induced injury at different doses can thus be quantified by loss of fluorescent staining in HCs (Ou et al., 2010). Therefore, at 5 dpf, larvae were transferred to 12-well plates (5–11 fish per well, 1 well per condition) containing the relevant compound or combination diluted with ICS water, or ICS water alone or high-dose neomycin to serve as controls. Unless otherwise noted, fish were exposed to drug (either an individual compound or a pairwise combination of compounds) for 4 h, as a balance between longer exposure times for accommodating varied kill kinetics and shorter exposure times for technical tractability. Treatments with multiple drugs involved co-administering a prepared solution consisting of both drugs with defined concentrations in ICS water. Fish were then washed in fresh ICS water before being transferred to new 12-well plates containing a 2 µM solution of YO-PRO-1 for 20 min, before being washed in fresh ICS water, anesthetized with 675 µM tricaine (Table 1), and transferred to 96-well plates for imaging (1 fish per well, 60 fish per plate due to specifications of the microscope).

**FIGURE 1:**
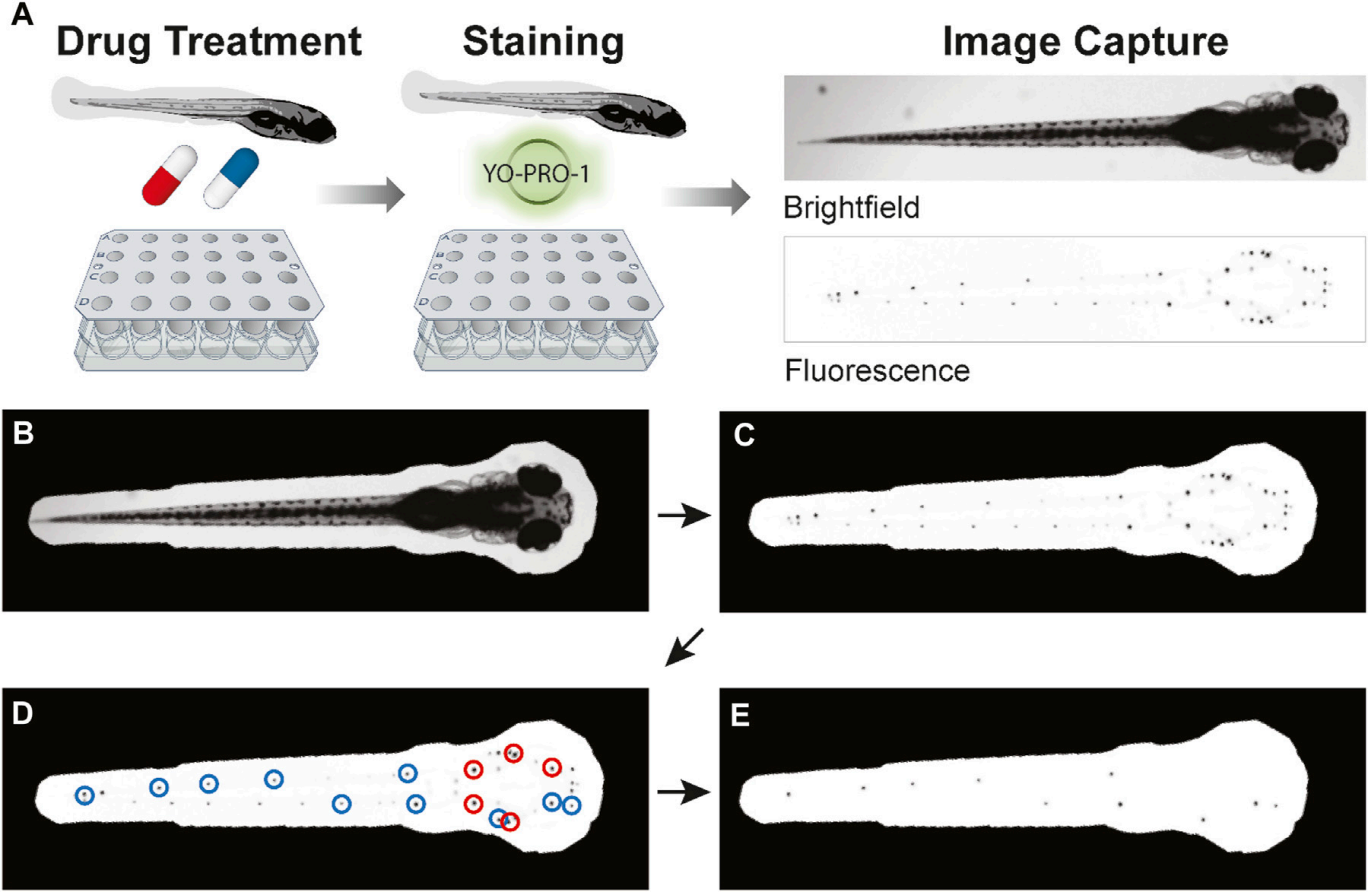
Overview of PEPITA workflow. **(A)** Zebrafish at 5dpf are placed into custom-made strainers in multi-well plates to be drug-treated and stained. After drug treatment for 4 h and YO-PRO-1 staining for 20 min, fish are anesthetized and imaged in brightfield and fluorescent channels. The resulting images are then quantified and analyzed. **(B–E)** An overview of PEPITA’s processing steps for quantifying whole-organism zebrafish image data. PEPITA accepts as input brightfield and fluorescence images of each organism. An additional fluorescence channel with no fluorophore present can also be optionally supplied, in which case PEPITA will use it as a baseline to cancel out autofluorescence in the image of fluorescently labeled neuromasts. **(B)** PEPITA creates a mask by automatically locating the larva by contrast, size, and shape from the brightfield image (which can be overridden manually when necessary). **(C)** This mask is applied to the fluorescence image in order to identify the points of interest. **(D)** PEPITA next identifies the 15 brightest local maxima within the masked region, excludes the top five (marked here in red), and creates a second mask obscuring everything except small circles (with a radius of 8 pixels by default) around the other ten puncta (marked here in blue). **(E)** This second mask is then reapplied to the fluorescence image, and the unobscured pixel values that exceed background level are summed to yield the raw fluorescence score for the given larva.

To facilitate the transfer of fish between exposures to drugs, fresh ICS, and YO-PRO-1 solutions, we developed custom single-use multi-well baskets (Figure 1A), inspired by the baskets described by Thisse and colleagues (Thisse and Thisse, 2008). We designed the frame for these baskets (https://www.thingiverse.com/thing:5676024), which we printed with a Dremel Digilab 3D45 3-D printer to provide a single point of manipulation for the whole plate. Nylon mesh was melted to this frame as previously described to form a basket for each well of the relevant multi-well plate (Thisse and Thisse, 2008). Each multi-well basket was disinfected with 10% bleach soak prior to use.

### 2.3 Imaging and ototoxicity quantification

Fluorescence microscopy was performed using a Keyence BZ-X800 microscope imaging system, which enables high-throughput semi-automated imaging of 96-well plates. Using a GFP filter (525/50 nm emission, 470/40 nm excitation), each YO-PRO-1-stained fish in the 96-well plate was imaged under a ×2 objective to capture the whole organism in brightfield, green fluorescence, and red fluorescence channels.

The resulting images were analyzed for dose-response and drug interaction characteristics with the PEPITA software package, which can be found on GitHub (https://github.com/ma-lab-cgidr/PEPITA-tools). Images did not have to be consistent in exposure or aperture used, as values were postprocessed, making use of the fact that received signal is directly proportional to the exposure time and inversely proportional to the square of the aperture *f*-stop (42, 2019). Each image was masked using automated object detection to include only the fish itself. Manual masks were created on occasion for avoidance of fluorescent contamination, adjusted segmenting for *myo6b::gfp* fish to remove inner ear HC fluorescence, or exclusion of dead or damaged fish. Each fish was then adjusted for autofluorescence using the fluorophore-free red channel and scored based on the sum of green fluorescence pixel values surrounding 10 of the top 15 brightest pixels in the masked image (Figures 1B–E). These scores were standardized by comparison with the median score of wells containing untreated fish and a score representing no remaining HC fluorescence, both derived for each plate. The standardized relative fluorescence unit (RFU) scores were fitted against a log logistic model, as described previously (Ritz, 2010), to estimate dose response properties (e.g., EC_50_, the effective concentration of eliciting 50% of maximal HC damage).

To prepare the fish for confocal microscopy, fish were fixed in 2% paraformaldehyde for 1 hour at room temperature as described previously (Philip et al., 2018), before being transferred to fluoromount and mounted between two glass coverslips. To avoid crushing the fish, a dot of nail polish was placed on each corner of the coverslip so that the top coverslip sat slightly above the bottom coverslip where the fish was mounted. Fluorescent confocal microscopy was performed using a Leica Stellaris 8 microscope. Cranial lateral line neuromasts of each fish were imaged under an HC PL APO CS2 40x/1.30 oil objective with a 405 nm laser and 541/47 emission filter. Images were taken as z-stacks in 0.66 µm steps. Stacks were transformed into average intensity projections and prepared for visual analysis in ImageJ.

For hair cell survival quantification from individual neuromasts, trained observers counted surviving hair cells in confocal images of neuromasts of larvae that had been treated with various doses of neomycin. Two to eight neuromasts were scored per condition, and counts for each neuromast were averaged between two scorers. These counts were divided by previously characterized mean hair cell counts (Harris et al., 2003), matched by anatomical position, to yield a position-scaled measure of HCs present in each neuromast. These position-scaled values were then converted to estimated percent remaining HCs by dividing by the average position-scaled value for untreated neuromasts of the same strain and experiment.

### 2.4 Aminoglycoside uptake quantification

To further explore the mechanism of macrolide and aminoglycoside antagonism, we fluorescently labeled the aminoglycosides neomycin and gentamicin with Texas Red-X succinimidyl ester to track HC uptake with and without macrolide treatment (Table 1). To avoid breakdown of fluorescent vital dyes over time, we used larvae with the transgene *Tg(myo6b:EGFP)*; *myo6b::gfp*, that expressed GFP in their HCs. Drug-induced injury was quantified by loss of fluorescence in HCs. Fish were exposed to the fluorescently-labeled drug with or without macrolide co-administration for 30 min, before being washed in fresh ICS water and anesthetized with 675 µM tricaine. Five fish per condition were transferred to 96-well plates for imaging, as per the drug response testing experiments, while another five were euthanized on ice for 20 min, then fixed in 2% paraformaldehyde for 1 hour at room temperature, to prepare for confocal microscopy. Samples were prepared and GFP fluorescence was acquired following the same confocal imaging procedure as described in Section 2.3; TexasRed fluorescence was acquired with a 595 nm laser and 675/75 emission filter.

For the fish transferred to 96-well plates for imaging, fluorescence microscopy was performed using the Keyence BZ-X800. Using the GFP filter and a TexasRed Filter (630/75 nm emission, 560/40 nm excitation), each *myo6b::gfp* fish was imaged under a ×2 objective to capture the whole organism in brightfield, green, and red fluorescence channels. Each fish was then imaged under a ×40 objective with z-stacking to track the uptake of the fluorescently labeled aminoglycoside into the HCs in the head of the fish.

### 2.5 FM1-43 uptake quantification

FM1-43 experiments were performed with *myo6b::gfp* fish, raised to 5 dpf as above. Fish were exposed to drug conditions for 30 min, with 4 µM FM1-43 stain added into the drug treatment solution at minute 29, for a 1-min co-treatment stain exposure followed by simultaneous washout of all drugs and stain in fish water and anesthesia in 675 µM tricaine (selection of staining concentration and exposure duration was informed by (Hernandez et al., 2006; Van Trump et al., 2010; Thomas et al., 2013; Monroe et al., 2016; Hailey et al., 2017; Majumder et al., 2017; Rocha-Sanchez et al., 2018; Stawicki et al., 2019; Parkinson and Stawicki, 2021; Lee et al., 2022; Derudas et al., 2023; Schrauwen et al., 2023)). Four to seven fish per condition were transferred to 96-well plates for imaging. Using the Keyence BZ-X800 with GFP filter and a custom filter (605/70 nm emission, 470/40 nm excitation), each fish was imaged with a ×2 objective in brightfield and green and red fluorescence channels as above. FM1-43 uptake was validated using benzamil, a known uptake inhibitor (Rusch et al., 1994).

### 2.6 Lateral line hair cell intracellular calcium quantification

Calcium imaging was performed using an inverted Marianas spinning disk confocal system (Intelligent Imaging Innovations, 3i) with an Evolve 10 MHz EMCCD camera (Photometrics) and a Zeiss C-Apochromat 63x/1.2 numerical aperture water objective. Larvae were 5-6 dpf at the time of imaging. Larvae were first anesthetized in embryo media containing 800 µM tricaine, then stabilized on their sides under a harp, so that posterior neuromasts were exposed to the surrounding media. Imaging was performed at ambient temperature, approximately 25°C. GCaMP fluorescence was acquired with a 488 nm laser and 535/30 emission filter, with an exposure time of 100 ms, and RGECO fluorescence was acquired with a 561 nm laser and 617/73 emission filter and exposure time of 200 ms. Images were taken every 2 minutes as z-stacks through neuromasts in 2 µm steps. Neomycin and azithromycin were dissolved as a 4X stock in EM (described in Section 2.1: Zebrafish Husbandry), then added to the bath to achieve final working concentration after a 10-min baseline. Imaging of neuromasts continued for 50 min. Images were analyzed in ImageJ. Mean fluorescence intensity was measured for ROIs of individual randomly selected HCs (2–3 HCs per NM, see example ROI in Figure 4). For each HC, values were normalized to the average of baseline using Microsoft Excel.

### 2.7 Bacterial drug response testing

For bacterial drug interaction testing, bacterial strains *Staphylococcus aureus* ATCC 29213, *Escherichia coli* ATCC 25922, were grown from frozen stocks on 5% sheep blood agar for 16 h at 37°C, and *Mycobacterium* abscessus ATCC 19977 was grown from frozen stocks on Middlebrook 7H10 agar for 3 days at 37°C (CLSI, 2020). On the day of inoculation, serial dilutions of the appropriate drugs were performed in separate plastic troughs and then combined in a clear, round bottom, cell-culture treated 96 well plate. Macrolide serial dilutions ascended in concentration row-wise (left to right) and aminoglycoside serial dilutions ascended in concentration column-wise (top to bottom). A bacterial inoculum of 0.5 (±10%) McFarland units was prepared in sterile saline and then diluted 1:100 in the appropriate media. The drug dilution plate was inoculated with 100 µL of the bacterial inoculum, and the plate was sealed and incubated at 37°C for 18 h to 3 days, depending on the strain. After incubation, plates were read on an indirect mirror box by eye for inhibition of all visible growth and with a SpectraMax i3x microplate reader for absorbance at 600 nm.

### 2.8 Drug interaction quantification

We quantified drug interactions with the excess over Bliss (EOB) metric (Berenbaum, 1989). Given a combination of drugs A and B, EOB was calculated for each individual well exposed to doses of both drugs based on monotherapy and combination responses with the formula $EOB=R_{a}\times R_{b}-R_{ab}$ (1) (Berenbaum, 1989). Wells where the expected response ( $R_{a}\times R_{b}$ ) and observed response ( $R_{ab}$ ) were both less than 10% or greater than 90% were excluded from aggregation to reduce the effect of our choice of dose range. The remaining wells were summarized by averaging into a composite interaction score for the combination; we refer to this metric as the “windowed excess over Bliss” (wEOB).

### 2.9 Statistical analysis

Error bars, for both point estimates and line plots, indicate 95% confidence intervals, calculated by bootstrapping, as a nonparametric measure of uncertainty (DiCiccio and Efron, 1996). All zebrafish fluorescence data are included except for dead fish, fish too out of plane to view enough neuromasts for robust analysis, and experiments in which untreated controls are dimmer than several conditions that should have reduced HC fluorescence based on past data or literature. All bacterial data are included except for experiments in which uninhibited bacterial growth controls are less than the 60th percentile of measured wells or media only controls are greater than the 40th percentile of measured wells. Values plotted with no error bars depict individual measurements for the given conditions; when depicted with error bars, points represent the arithmetic mean of all valid measurements. For dose-response curve shift values, effective doses are estimated by interpolation on a logarithmic scale, and confidence intervals are estimated similarly based on bootstrapped point estimates. Shift *p*-values are calculated by extra sum-of-squares F test using the drc package in R (Ritz et al., 2015; Team, 2021). Elsewhere, *p*-values are calculated with Welch’s unequal variances *t*-test (West, 2021). Multiple testing is adjusted for with Benjamini-Hochberg FDR correction when noted (Benjamini and Hochberg, 1995).

## 3 Results

### 3.1 High-throughput quantification of lateral line damage and ototoxic drug interactions with PEPITA

We have developed PEPITA, a novel, semi-automated workflow for streamlined quantification of ototoxicity in zebrafish (Figure 1). The PEPITA workflow builds upon previous work that demonstrated that damage to zebrafish larval lateral line HCs could be quantified by evaluating the brightness intensity of fluorescently stained HCs using vital dyes such as YO-PRO-1 (Harris et al., 2003; Owens et al., 2008; Ou et al., 2009; Coffin et al., 2010; Thomas et al., 2015). Our platform enumerates relative residual brightness of YO-PRO-1-stained HCs after exposure of larvae to doses of individual or combinations of drugs. We quantify this remaining HC fluorescence as a proxy measure that is inversely proportional to the damage elicited by the ototoxic treatments.

**FIGURE 2:**
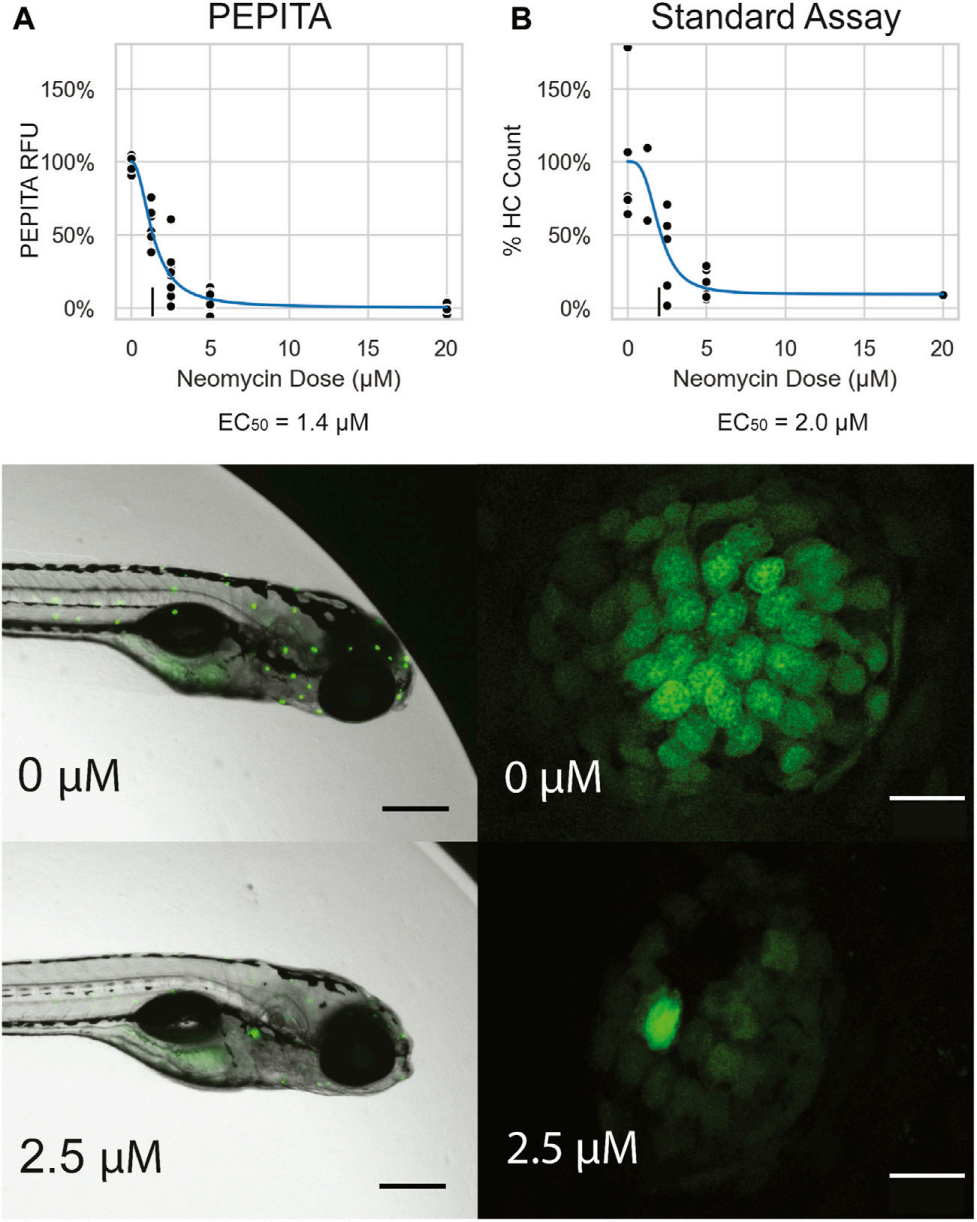
Characterization of single drug dose response with PEPITA. **(A)** Images and quantification of neomycin dose response from a representative experiment, using PEPITA **(A)** and the standard approach of counting HCs from individual neuromasts **(B)**. All fish featured in this figure were treated and characterized in the same experiment. **Top:** dose-response curve generated by the PEPITA workflow with relative fluorescence units (RFU) (EC_50_ = 1.4 μM, **left**) shows similar properties to the dose response curve derived by enumerating normalized HC counts from individual neuromasts using procedure described in Section 2.3 (EC_50_ = 2.0 μM, **right**). **(A) middle:** representative image of a fish exposed to no drug, which is used for PEPITA quantification; **(B) middle:** representative image of an individual neuromast from a fish that was exposed to no drug, used for HC counting. **(A) bottom**: representative image of a fish exposed to 2.5 μM NEO, which is used for PEPITA quantification; **(B) bottom**: representative image of an individual neuromast from a fish that was exposed to 2.5 μM NEO, used for HC counting. Note that PEPITA uses the image of the whole fish for quantification, whereas the images have been cropped in these panels to faciliate visual inspection of the stained neuromasts. Scale bars in fish images represent 300 μm, and scale bars in neuromast images represent 10 μm.

PEPITA enables quantification of hundreds of individual YO-PRO-1-stained fish per experiment. This high-throughput quantification of ototoxic damage in zebrafish larvae enables us to perform granular, reproducible characterizations of ototoxic dose-response curves for each drug of interest (Figure 2; Supplementary Figures S1, S2).

To compare PEPITA’s ototoxic dose response characterization with the gold-standard approach of quantifying ototoxic damage (Chiu et al., 2008; Owens et al., 2008; Philip et al., 2018), we also performed side-by-side characterizations with manual HC counting of individual neuromasts in both YO-PRO-1–stained AB fish and *myo6b::gfp* fish (Figure 2, Supplementary Figure S1). Importantly, we find that dose response parameters derived from PEPITA correspond closely with estimates calculated by manual HC counting in both fish lines (Figure 2, Supplementary Figure S1). We also find that AB larvae stained with YO-PRO-1 and *myo6b::gfp* larvae present very similar neuromast appearance when untreated, at both high and low magnification, and decrease in fluorescence in a comparable way when treated with increasing doses of ototoxic drug. The two strains exhibit differences in doses necessary to achieve comparable levels of fluorescence inhibition. For instance, in our hands, the neomycin dose required to elicit 50% of maximal HC damage (EC_50_, see Methods for details) was 20 μM in *myo6b::gfp* fish and 2.5 μM in AB fish stained with YO-PRO-1 (Figure 2, Supplementary Figure S1). Even in *myo6b::gfp* fish, this is lower than what has been observed previously, likely due to salinity differences, especially in calcium and magnesium, between our fish water and that of other investigators (Coffin et al., 2009; Linbo et al., 2009).

The shift between YO-PRO-1–stained AB and *myo6b::gfp* fish might potentially be due to a combination of factors, including differences in genetic background, an altered susceptibility to HC damage conferred by GFP (Monroe et al., 2016), and/or a subtle difference in the target of the assays’ direct measurement: HC survival (*myo6b::gfp*) *versus* HC functionality (YO-PRO-1–stained AB). The *myo6b::gfp* fish express GFP in HC cytoplasm on the myosin 6 promoter, so stresses should not affect cell fluorescence up to the point of membrane rupture. In contrast, AB fish are stained with YO-PRO-1 after drug treatment, and this dye is taken up by the cell and fluoresces when it binds DNA (Santos et al., 2006). This means that, in addition to cell death, changes in uptake, trafficking, and DNA organization—all important signs of HC functionality that could be impaired even with mild, sublethal ototoxic effects—can affect fluorescence (Chiu et al., 2008). Aminoglycosides cause damage at even relatively low doses, and at sufficient doses induce cell death by various pathways (Coffin et al., 2013). While HC death would result in reduced fluorescent signal for both *myo6b::gfp* and AB fish, sub-lethal HC damage affecting cell functionality would only reduce fluorescence for YO-PRO-1–stained AB fish. This implies two important caveats with regard to screening individual compounds with PEPITA: 1) ototoxic dose response profiles for individual drug treatments may vary depending on the genetic background of larvae tested, and 2) compounds that block YO-PRO-1 uptake without causing ototoxic damage may yield false positive indications for HC death when using AB fish stained with YO-PRO-1 alone, as we have observed with benzamil (Supplementary Figure S3). Complementary characterization with *myo6b::gfp* or other fish lines with transgenically labeled HCs will help to resolve these false positives. While our post-processing is currently optimized for reading out AB staining data, the platform is flexible to the use of other transgenic or stained fish as well.

Despite these differences in the underlying realities being measured, and the resulting difference in effective concentrations from individual drug exposure, it is important to note that observed drug-drug interaction effects appear to be comparable. When detected, antagonism between drug pairs as quantified by windowed excess over Bliss (wEOB, see Methods for detailed description) is observed to equivalent degrees in both assays (Supplementary Figure S4).

### 3.2 Azithromycin broadly antagonizes aminoglycoside ototoxicity and confers otoprotection

**FIGURE 3:**
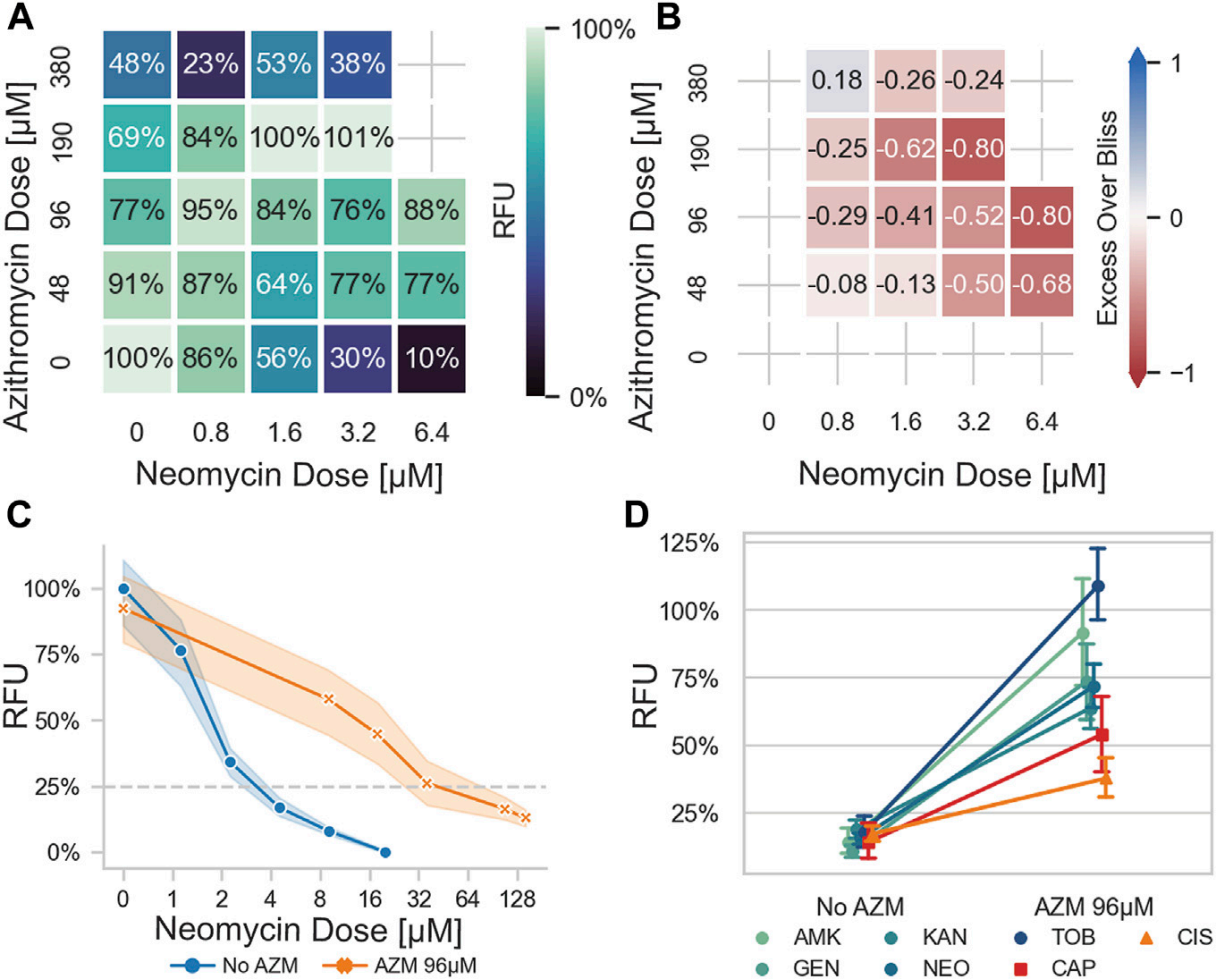
Azithromycin antagonizes aminoglycoside-induced ototoxicity in a zebrafish model. **(A)** Response seen when fish were exposed to increasing concentrations of azithromycin (AZM) and neomycin (NEO) in checkerboard format, as quantified by PEPITA in relative fluorescence units (RFU). Fish exposed to a combination of AZM and NEO experienced less ototoxic damage than those exposed to NEO alone, at all significantly ototoxic doses of NEO (i.e., ≥1.6 μM) and AZM doses up through 190 μM. In an extreme case, a dose of 6.4 μM NEO, causing 90% HC damage (10% RFU), is reduced to minimal damage (88% RFU) by the addition of 96 μM AZM. **(B)** excess over Bliss values calculated for the previous checkerboard data: positive numbers indicate synergy, negative numbers antagonism. The trend of reduced damage seen in the checkerboard translates to consistent antagonism, with an overall wEOB of −0.36 for this experiment. **(C)** Comparison of lateral line HC ototoxic dose response elicited by NEO with vs. without AZM co-administration (96 μM). **(D)** Comparison of lateral line HC damage in response to ototoxic drug exposure with vs. without AZM co-administration (96 μM). Drugs tested: AMK = amikacin, GEN = gentamicin, KAN = kanamycin, NEO = neomycin, TOB = tobramycin, CAP = capreomycin, CIS = cisplatin. Doses of drugs tested were selected to elicit 70%–95% hair cell damage in the absence of AZM co-administration. Aminoglycosides as a group are more antagonized than the non-aminoglycoside drugs tested (*p* < 0.0001).

The high-throughput nature of PEPITA enables screening of ototoxic interactions experienced by fish by concomitant administration of combinations of drug treatments. In an initial pairwise screen of neomycin (NEO) against 13 compounds reported in the literature to impact HC survival and function (Rusch et al., 1994; Himeno et al., 2002; Rybak and Whitworth, 2005; Granowitz and Brown, 2008; Crumling et al., 2017; Lanvers-Kaminsky et al., 2017; Sheth et al., 2017), we found that azithromycin (AZM) was one of the strongest antagonizing compounds to ototoxicity (Supplementary Figure S5). To study the impact of this antagonism on the extent of otoprotection conferred by co-administration of AZM, we compared the extent of HC damage at varying doses of NEO exposure, with and without co-treatment with varying doses of AZM (Figures 3A–C). Although administration of high doses of AZM confers ototoxic damage (exposure to 380 µM AZM for 4 h resulted in 48% residual HC fluorescence in YO-PRO-1-stained larvae, as quantified by relative fluorescence units (RFU), Figure 3A), co-administration of 96 µM of azithromycin rescued HC viability at concentrations of neomycin that were otherwise toxic to HC (exposure to 6.4 µM NEO for 4 h resulted in 10% RFU in the absence of AZM, but 88% RFU when co-administered with 96 µM AZM, Figure 3A), and shifted the dose of neomycin needed to elicit 75% maximal HC damage (EC_75_) by 13-fold (3.3 µM–42 μM, Figure 3C). This corresponded to wEOB score of −0.36, indicating strong antagonism (Figure 3B). This antagonism was observable in both AB and *myo6b::gfp* experiments (Supplementary Figure S4).

We wondered at the extent to which the antagonistic interaction between AZM and NEO observed with ototoxic response generalized to other aminoglycosides. We therefore tested ototoxic interactions between AZM and 5 aminoglycosides (neomycin (NEO), amikacin (AMK), gentamicin (GEN), tobramycin (TOB), and kanamycin (KAN)) (Figure 3D, Supplementary Figure S6; Table 1). Among interactions with AZM, co-administration with each of the 5 aminoglycosides exhibited otoprotective antagonistic interactions. For doses of each aminoglycoside that reduced functional HC survival by at least 70% when treated alone, co-administration with AZM resulted in 62%pt. protection in HC survival on average (improving from 15% survival to 77% survival on average; Figure 3D). In comparing the ototoxic HC damage dose response, the EC_75_ of each aminoglycoside shifted at least 8-fold with AZM co-administration (Table 2, Supplementary Figure S7).

**TABLE 2:** Magnitude of EC75 shift when ototoxic drugs are co-administered with a macrolide as compared to administered alone. The aminoglycosides display large shifts with high levels of statistical significance; CAP and CIS show lower shifts that border on significance. The *p*-values listed represent the probability that the dose-response curve shift we observed occurred solely due to chance, as quantified by an extra sum-of-squares F test.

Macrolide	Ototoxin	EC75 shift	Shift *p*-value
Azithromycin (AZM)	Amikacin (AMK)	8-fold	0.0003
	Gentamicin (GEN)	13-fold	<0.0001
	Kanamycin (KAN)	>16-fold	<0.0001
	Neomycin (NEO)	13-fold	<0.0001
	Tobramycin (TOB)	>16-fold	<0.0001
	Capreomycin (CAP)	2.8-fold	0.057
	Cisplatin (CIS)	1.8-fold	0.048
Erythromycin (ERY)	Neomycin (NEO)	8-fold	<0.0001

We also tested the impact of AZM co-administration on protection of ototoxicity with neomycin (NEO) and gentamicin (GEN) at two different time points: 1 h (acute) and 4 h (extended) of treatment. These timepoints were selected because they had previously been shown to convey differences in protection between NEO and GEN when co-administered with different cell death inhibitor compounds (Coffin et al., 2013). We found that AZM elicited significant protection against both NEO and GEN after 1 h and 4 h of treatment (for all conditions in which monotherapy induced a significant level of damage, AZM co-treatment conferred a significant level of otoprotection, Supplementary Figures S8A–D).

### 3.3 Azithromycin-induced otoprotective antagonism is significantly pronounced with aminoglycosides

To evaluate the specificity of AZM-mediated otoprotective antagonism, we measured the ototoxic interactions of AZM coadministration with non-aminoglycoside drugs that convey ototoxicity clinically and share similarities in their modes of toxicity and action: cisplatin and capreomycin (Table 1). Cisplatin (CIS) was selected because of the similarity of the drug uptake mechanism in HCs relative to aminoglycosides (both sets of drugs require mechanoelectrical transduction (MET) channel activity for HC uptake) (Barrallo-Gimeno and Llorens, 2022). Capreomycin (CAP) was selected because it is an ototoxic antibiotic with a similar mode of action compared to aminoglycosides, but has a chemical structure with distinct chemical properties (Akbergenov et al., 2011; Krause et al., 2016; Rybak et al., 2021).

We found that AZM co-administration conferred significant but less pronounced protection from CAP-associated damage, (Figure 3D, Supplementary Figure S7). Exposure to 25 μM CAP yielded 90% HC inhibition in the absence of azithromycin (CI 83%–96%) and 60% HC inhibition in the presence of azithromycin co-administration (CI 45%–76%) (Figure 3D). In comparing the ototoxic HC damage dose response, the EC_75_ of CAP shifted 2.8-fold (Table 2, Supplementary Figure S7). Notably, this protection only seems to be present in highly damaging doses of CAP as the CAP EC_50_ shift was not significant (1.9-fold; CI −1.5–3.8 fold).

Similarly, AZM co-administration conferred significant but modest protection against damage caused by higher doses of CIS. Exposure to 600 μM CIS yielded 81% HC inhibition (CI 78%–83%) in the absence of AZM and 57% HC inhibition (CI 46%–68%) in the presence of AZM co-administration. As with CAP, AZM co-administration conferred stronger protection under conditions of high HC damage (EC_75_ of CIS shifted 1.8-fold (CI 1.4 to 2.2-fold)) than lower HC damage (EC_50_ of CIS shifted 1.4-fold, CI -1.9 to 2.8-fold) (Table 2, Supplementary Figure S7).

### 3.4 Macrolide antibiotics broadly confer antagonistic otoprotection against aminoglycoside ototoxicity

To evaluate the extent to which AZM’s otoprotective antagonism against aminoglycosides extends to other drugs in its class, we also tested two other commonly used macrolide antibiotics, erythromycin (ERY) and clarithromycin (CLM), on drug interactions with aminoglycosides. Based on our preliminary experiments, we found antagonistic interactions between CLM and each of AMK, GEN, KAN, and NEO (Supplementary Figure S6); however, we estimated that the optimally protective dose of CLM would be higher than the solubility threshold in water, so we chose to focus our characterization on ERY. We found that ERY also conveyed antagonistic otoprotection broadly against aminoglycoside ototoxicity during co-administration, although the most otoprotective dose of ERY was significantly higher than AZM (Supplementary Figure S7). The EC_75_ of NEO shifted 8-fold (CI 4.0 to 17-fold) with co-administration of ERY (Table 2, Supplementary Figure S7).

### 3.5 Macrolides interfere with aminoglycoside uptake into hair cells

Given that macrolide co-administration appeared to confer comparable otoprotection against neomycin after both acute (1-h) and extended (4-h) exposure, we hypothesized that the mechanism of macrolide-associated protection involved influencing drug uptake.

**FIGURE 4:**
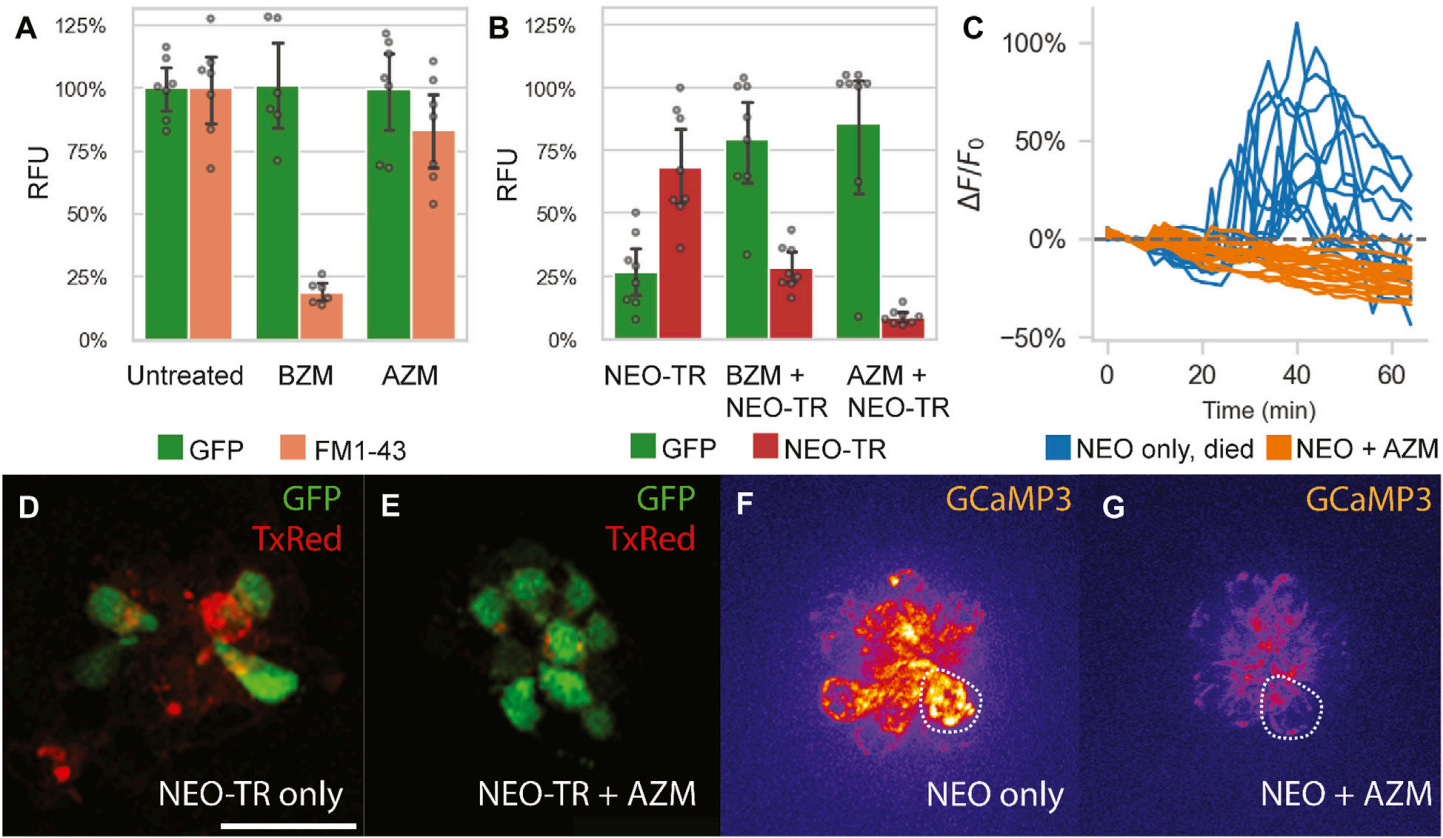
Impact of azithromycin and neomycin treatment on hair cell function. **(A)** AZM (190 µM) does not significantly hinder FM1-43 uptake into lateral line HCs (*p* = 0.15), as quantified by PEPITA. This suggests that AZM does not inhibit MET function. In contrast, administration of the MET inhibitor benzamil (BZM; 50 µM) as a positive control does significantly reduce FM1-43 uptake (*p* < 0.0001). **(B)** Effect of AZM (190 µM) co-administration on accumulation of TexasRed-conjugated neomycin (NEO-TR, 50 μM exposure) within *myo6b::gfp* lateral line HCs, as quantified by PEPITA. BZM (50 µM) co-administration with NEO was also evaluated as a positive control, and both conferred inhibition of NEO-TR uptake into the hair cells (BZM, *p* = 0.001; AZM, *p* = 0.0001). **(D and E)** Representative fluorescence microscopy images of the colocalization of NEO-TR (TexasRed (TxRed), red) with GFP-expressing HCs (GFP, green), treated with NEO-TR alone **(D)** or with both NEO-TR and AZM **(E)** for 30 min, after washout, not used for quantification of NEO-TR accumulation or GFP fluorescence levels. **(C)** Quantification of mitochondrial Ca^2+^ levels in individual HCs in response to NEO treatment alone (blue) or NEO plus AZM co-administration (orange), as measured by *mitoGCaMP3* fluorescence signal. Drugs were administered at t = 10 min; **(F and G)** Fluorescence microscopy images of *mitoGCaMP3* neuromasts at 30 min post drug administration, treated with NEO alone **(F)** or with both NEO and AZM **(G)**. Scale bar for panels (**D–G)** represents 20 µm. The dashed shapes in panels **F** and **G** depict example regions of interest (ROI) used to quantify fluorescence signal.

To test the effect of macrolide co-administration on aminoglycoside uptake, we adopted two complementary approaches: 1) measuring the effect of AZM administration on MET channel activity via HC uptake of FM1-43, a dye whose transport is mediated by MET channel (Gale et al., 2001) (Figure 4A); and 2) testing the effect of AZM co-administration on HC uptake of fluorescently-conjugated neomycin (Figure 4B).

We measured accumulation of FM1-43 in lateral line HCs in the presence vs. absence of AZM (190 µM) in *myo6b::gfp* larvae. AZM administration for 30 min prior to and during FM1-43 administration did not significantly change FM1-43 accumulation in HCs, whereas administration with the positive control, benzamil (BZM, 50 μM, a known MET channel blocker (Rusch et al., 1994)), for the same period showed significant inhibition of FM1-43 uptake (Figure 4A). These data suggest that MET channel activity is not impaired by AZM administration.

We also measured accumulation of TexasRed-conjugated neomycin (NEO-TR, 50 µM) in lateral line HCs in the presence vs. absence of AZM (190 µM) in *myo6b::gfp* larvae. AZM co-administration yielded reduced HC accumulation of NEO-TR (indicated by reduced TexasRed labeling in HCs after 30 min of exposure to TexasRed-conjugated drug), which was concomitant with reduced HC damage (indicated by increased GFP labeling in the same HCs) (Figures 4B, D, E). These data suggest that neomycin uptake is inhibited by azithromycin co-administration.

Should macrolides block aminoglycoside uptake into HCs, we expected downstream cell signaling pathways associated with aminoglycoside exposure would also be blocked. For example, it has been previously shown that once taken up by HCs, NEO induces a large spike in mitochondrial calcium, and subsequently in cytoplasmic calcium in HCs that go on to die (Esterberg et al., 2014). To test if AZM blocks these effects, we measured HC mitochondrial and cytoplasmic calcium responses in response to both NEO alone (50 µM) and co-administration of NEO (50 µM) with AZM (190 µM) in *mitoGCaMP3; cytoRGECO* larvae. Consistent with the previous findings, we demonstrated NEO induced an increase in mitochondrial (Figures 4C, F, Supplementary Figure S9; *n* = 4 fish, 9 neuromasts) and cytoplasmic calcium in HCs that went on to die (66% of those analyzed). Co-administration of NEO with AZM, however, blocked increases in mitochondrial and cytoplasmic calcium (Figures 4C, G, Supplementary Figure S9; *n* = 5 fish, 10 neuromasts). Although we occasionally observed increases in mitochondrial calcium and low levels of cell death with AZM administration alone (Supplementary Figure S9), there was no cell death observed with NEO and AZM co-administration. These results suggest that AZM co-administration and subsequent NEO uptake inhibition further blocks the downstream signaling pathways associated with NEO-induced death, namely, changes in HC calcium handling.

### 3.6 Macrolides do not antagonize antimicrobial activity of aminoglycosides

**FIGURE 5:**
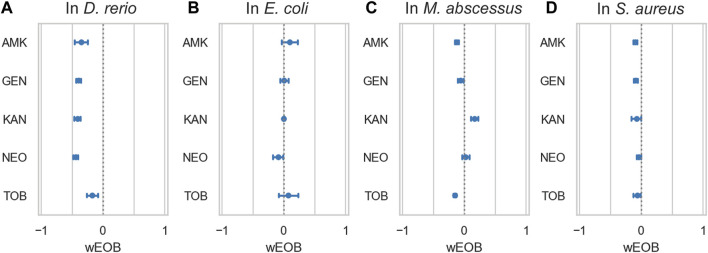
Azithromycin and aminoglycosides do not interact antagonistically in bacteria. **(A)** depicts ototoxic interactions in zebrafish as measured by PEPITA, while the subsequent three panels depict interactions as measured by checkerboard assay with the given bacterium, respectively: **(B)** *E. coli*, **(C)** *M. abscessus*, and **(D)** *S. aureus*. The *x*-axis quantifies the windowed excess over Bliss metric (wEOB), which measures drug interactions with negative numbers indicating antagonism and positive numbers indicating synergy. The *y*-axis contains 5 aminoglycoside antibiotics. None of the measured combinations yield a consistently antagonistic interaction in terms of the bacterial growth inhibition achieved, in contrast with the significant antagonistic protection AZM confers against damage induced by all of these drugs.

Given that both the macrolides and aminoglycosides that we tested are antibiotic agents, we investigated whether they antagonized each other’s antimicrobial activity during co-administration. Checkerboard assay experiments in *S. aureus, E. coli* and *Mycobacterium abscessus* (representing gram-positive, gram-negative, and atypical bacteria, respectively, Table 1), between AZM and 5 separate aminoglycosides (amikacin (AMK), gentamicin (GEN), kanamycin (KAN), neomycin (NEO), and tobramycin (TOB)) showed no pattern of antagonism (Figure 5). Average windowed excess over Bliss scores were 0.02 in *E coli*, −0.03 in *M. abscessus*, and −0.07 in *S. aureus*, suggesting on little interaction to even slight synergy. In comparing the interaction of these aminoglycosides with AZM, for AMK this represents a 31%pt. shift toward antagonism in lateral line HC damage as compared to inhibition of these bacterial species on average; for GEN 37%pt. toward antagonism, for KAN 45%pt. toward antagonism, for NEO 44%pt. toward antagonism, and for TOB 15%pt. toward antagonism. These values suggest that treating a bacterial infection with a regimen comprising AZM and any of these aminoglycosides would cause on average less ototoxicity relative to treatment with the aminoglycoside alone, without antagonizing the antimicrobial activity of the antibiotics.

## 4 Discussion

Given the multiplicity of potential drug-drug interactions and the complexity of their molecular consequences, anticipating the impacts of these interactions on toxicity outcomes is currently an important unresolved gap in the drug development process. High-throughput quantification of toxicity outcomes *in vivo* by tools such as PEPITA enables a unique lens into the treatment impact of combinations with toxicity-protective or toxicity-potentiating properties. By identifying candidate otoprotective multi-drug interventions, such as co-administration of macrolides with aminoglycosides, PEPITA can prioritize these combinations for follow-up translational preclinical studies. Since PEPITA can also quantify multiple drug treatment conditions in a single ototoxicity profiling experiment, PEPITA can also streamline investigation into the molecular mechanisms underlying toxicity and protection.

Previously published advances in automated quantification of ototoxicity in zebrafish have successfully streamlined the assessment of damage lateral line HCs from high-resolution images of YO-PRO-1-stained neuromast HCs taken by confocal microscopy (Philip et al., 2018). While this automated analysis approach facilitated faster interpretation of images once collected, the experimental workflow for preparing the treated and stained fish for imaging remained a laborious and time-consuming multi-day process, which also restricted the number of neuromasts captured to a relatively small number of representative neuromasts (7 per fish) on a relatively small number of fish. In contrast, the PEPITA workflow enables semi-automated image capture and analysis of live zebrafish larvae in a 96-well plate setting, without fixation or mounting of the larvae. By aggregating the staining information across multiple neuromasts from a ×2 objective image magnification image of each whole fish, the time required to acquire the necessary images for quantification is drastically reduced, typically lasting approximately 30 min per plate of 60 fish. Though some of the morphological details of the HCs are lost with a low-magnification image capture, PEPITA can capture more neuromasts per fish and more fish per experiment, which empowers us to achieve greater statistical power in assessing differences in ototoxic outcomes for each treatment.

Using PEPITA to characterize ototoxic drug-drug interaction outcomes, we have discovered a tissue-specific antagonistic interaction between macrolide antibiotics and aminoglycoside antibiotics that confers protection against aminoglycoside-induced injury to lateral line HCs in zebrafish larvae. Co-administration of macrolide antibiotics including azithromycin and erythromycin protected against lateral line HC damage induced by a panel of aminoglycosides. The successful identification of these protective interactions highlights the potential clinical translational utility of *in vivo* screening multidrug combinations for putative toxicity-protective interventions. Antagonism represents an understudied and underexploited modality of toxicity protection that might enable a novel avenue of repurposing clinically approved drugs towards toxicity-protective applications. Moreover, studying the molecular mechanisms underlying the protection of these antagonistic interactions might uncover new intervention targets that could protect against toxicity.

While clinical reports have noted observations of possible ototoxicity (Shim et al., 2024), the effect of macrolide antibiotics on HCs, including azithromycin, erythromycin, and clarithromycin, remain largely uncharacterized in the zebrafish lateral line model (Rybak et al., 2021). Previous investigations with the mTOR inhibitor macrolide, rapamycin, have found that rapamycin confers significant protection against cisplatin ototoxicity by activating autophagy (Pang et al., 2018). Macrolide antibiotics including azithromycin are known to inhibit autophagy (Renna et al., 2011; Moriya et al., 2013), and the protection they confer against cisplatin ototoxicity is modest. This therefore suggests that their mechanism of protection is distinct from rapamycin. Although we observed evidence of ototoxic HC inhibition with high-dose azithromycin alone (Supplementary Figure S10), our experiments with *myo6b::gfp* and *mitoGCaMP3* fish revealed that lower concentrations of macrolides inhibited aminoglycoside-induced HC toxicity when co-administered. This protective effect of macrolide antibiotics was similar when co-administered with aminoglycosides previously shown to have differences in their ototoxic mechanisms, such as gentamicin and neomycin (Coffin et al., 2013), though the protective effects of macrolides on other ototoxic drugs that share some commonalities in mechanism, including cisplatin and capreomycin, are different and substantially reduced.

In the case of neomycin, the otoprotection conferred by macrolide co-administration is correlated with reduction in aminoglycoside uptake by HCs but not with impaired MET channel activity. MET channel activity has previously been shown to be required for aminoglycoside ototoxicity (Alharazneh et al., 2011; Kenyon et al., 2021). Our data suggest that there exist MET channel-independent modalities of impairing neomycin uptake in HCs, resulting in protection. Our findings are also consistent with an aminoglycoside uptake model in which aminoglycoside uptake enters the stereocilia of HCs through a mechanism other than direct MET channel transport, but relies on MET channel activity for transport into the HC body. This model is consistent with studies that have implicated members of the transient receptor potential cation channels (TRPs) in aminoglycoside uptake (Karasawa et al., 2008; Lee et al., 2013; Wang et al., 2019); others have also speculated that non-selective cation channels, such as connexins, pannexins, and P2X channels may also be involved in aminoglycoside uptake (Jiang et al., 2017). Future studies examining the kinetics of macrolide protection at longer aminoglycoside exposure timepoints may help to dissect molecular mechanisms of action. Interestingly, the otoprotective macrolide antibiotics that we have identified in our study are also distinct in their chemical structures from known MET channel inhibitors that confer otoprotection (Supplementary Table S1); this suggests that the aminoglycoside uptake inhibition and subsequent protection conferred by macrolides represents a novel biochemical strategy of otoprotection.

In comparing the drug interaction outcomes measured from co-administration of macrolides and aminoglycosides in zebrafish lateral line HCs *versus* interactions from bacterial inhibition assays with multiple species, we find that the interactions of these drugs are not broadly correlated across tissues and across the drug classes. Notably, most of the drug combinations did not exhibit antagonism when inhibiting *E. coli, S. aureus, or M. abscessus*. In contextualizing this antimicrobial efficacy along with the pronounced protection of HCs, our data suggest that macrolide co-administration might shift the therapeutic index of aminoglycosides at least 8-fold, when considering zebrafish HC ototoxicity. The tissue specificity of drug interaction outcomes is consistent with previous reports that drug-drug interaction outcomes are a condition-specific phenotype (Cokol et al., 2018), and it also suggests that it may be possible to design regimens that improve therapeutic indexes by tuning antimicrobial interaction outcomes independently from host-targeting toxicity interaction outcomes. Given that differences exist between drug uptake and damage mechanisms in zebrafish lateral line HCs and adult mammalian inner ear HCs (Thomas et al., 2013), additional studies are warranted to evaluate the extent to which otoprotective antagonism between macrolides and aminoglycosides also occurs in mammals.

The proof-of-concept otoprotective antagonism discovered by the PEPITA platform raises hopes for identifying analogously protective combinatorial interventions that do not hinder on-target efficacy for other toxic drugs. Although PEPITA is currently optimized to quantify ototoxicity, the underlying platform and image analysis pipeline could be adapted to investigate interactions with other organ toxicities assayable in the zebrafish larval system.

The PEPITA-tools software package can be found on GitHub at https://github.com/ma-lab-cgidr/PEPITA-tools.

## Data Availability

The raw data supporting the conclusion of this article will be made available by the authors, without undue reservation.
