# Supplementary material for: In vivo screening for toxicity-modulating drug interactions identifies antagonism that protects against ototoxicity in zebrafish
Source: Front Pharmacol. 2024 Mar 7;15:1363545. doi: 10.3389/fphar.2024.1363545 (PMC10955247; doi:10.3389/fphar.2024.1363545)
Supplement: Supplementary file 1 [file DataSheet1.pdf]

Supplementary Material

1. Supplementary Figures

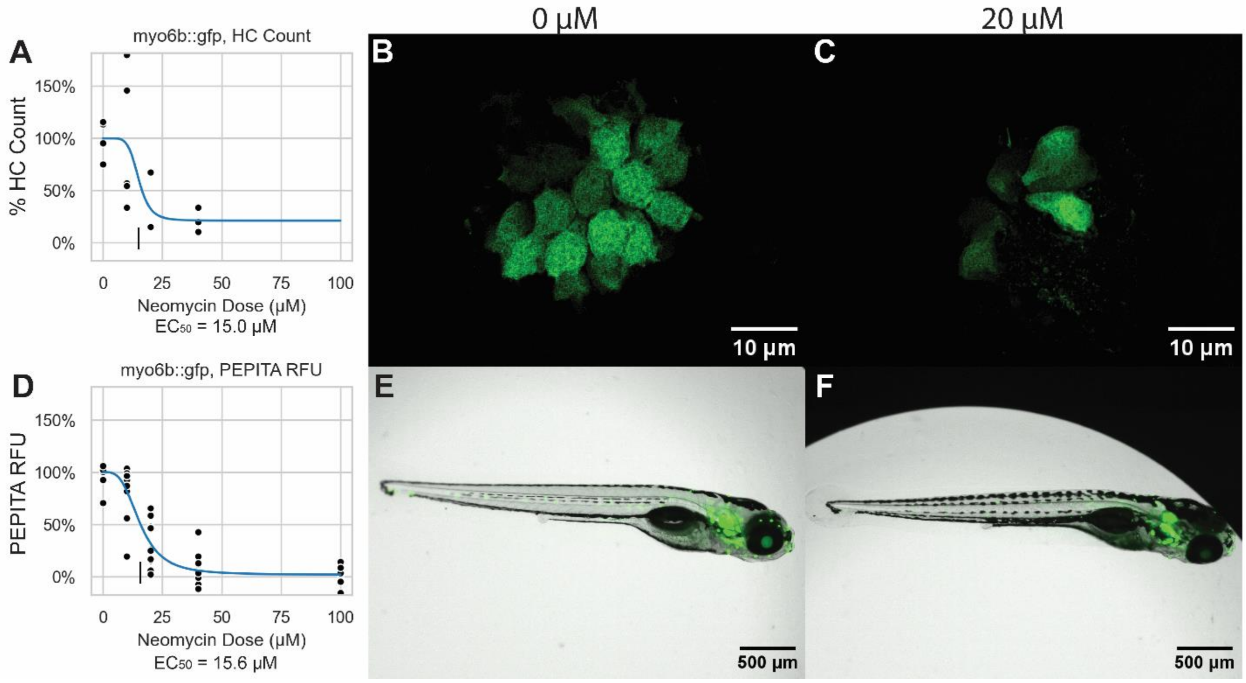

Figure S1 Neomycin dose response in *myo6b::gfp* fish. (A) Representative dose-response curve when exposed to increasing doses of NEO, as quantified by counting individual hair cells imaged by confocal microscopy. **(B)** Representative image of an undamaged neuromast. **(C)** Representative image of a damaged neuromast, resulting from exposure to 20 µM NEO. **(D)** Representative dose-response curve when exposed to increasing doses of NEO, as quantified by PEPITA. (**E,F)** Representative images of whole fish, like those PEPITA takes as input. **E** is untreated; **F** is treated with 20 µM NEO. The cluster of fluorescent inner-ear HCs in *myo6b::gfp* larvae requires manual masking for proper quantification with PEPITA.

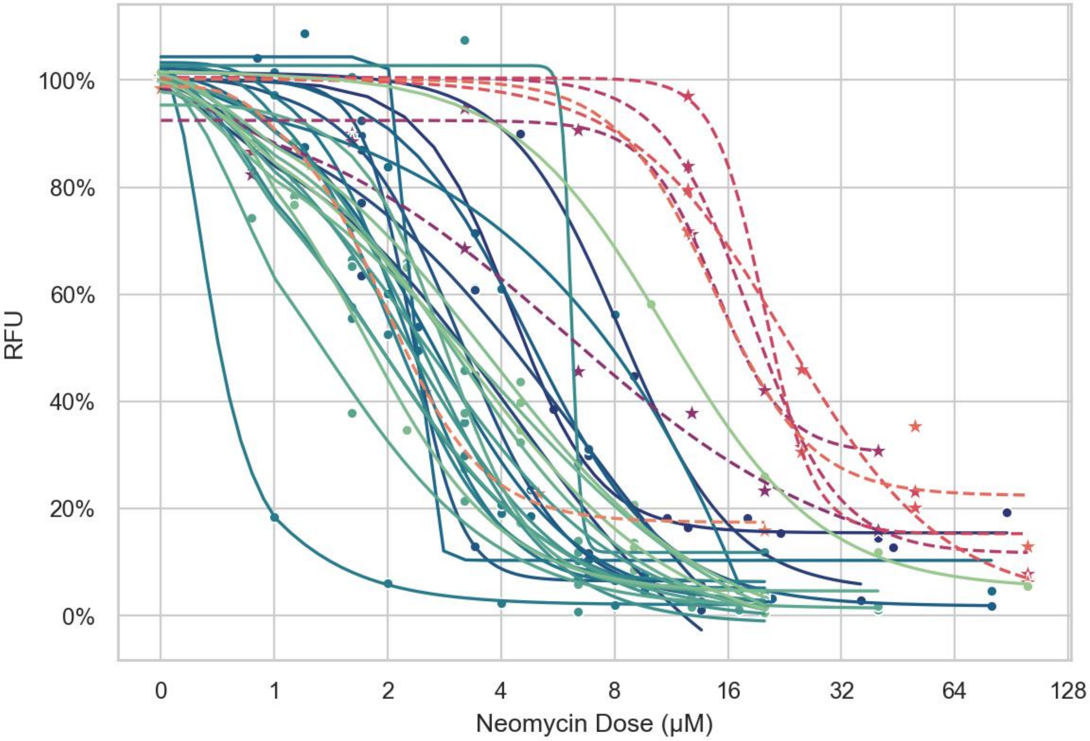

Figure S2. Overview of the variability in neomycin dose-response curves obtained by PEPITA. Points are measurements, lines are fitted log-logistic models; circles and solid lines represent AB fish stained with YO-PRO-1, stars and dashed lines represent *myo6b::gfp* fish. Colors represent separate experiments performed, categorized by strain: blues and greens represent AB fish, purples and reds represent *myo6b::gfp* fish.

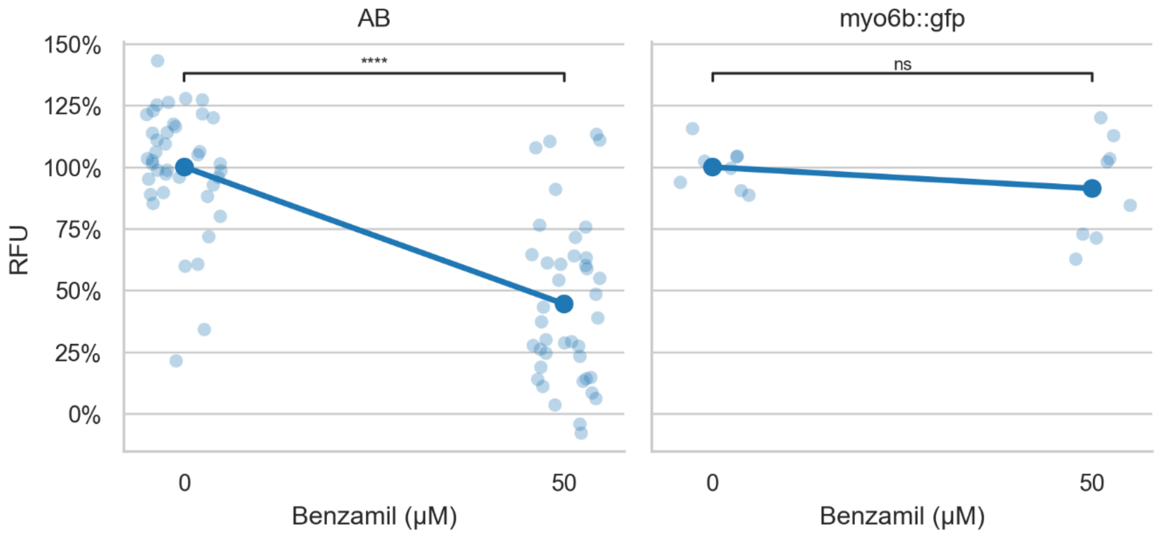

Figure S3. Effect of neuromast fluorescence intensity with benzamil treatment. (**Left**) RFU values generated from YO-PRO-1-stained AB fish. (**Right**) RFU generated from *myo6b::gfp* fish. **** = $p < 0.0001$, ns = not significant ($p > 0.05$).

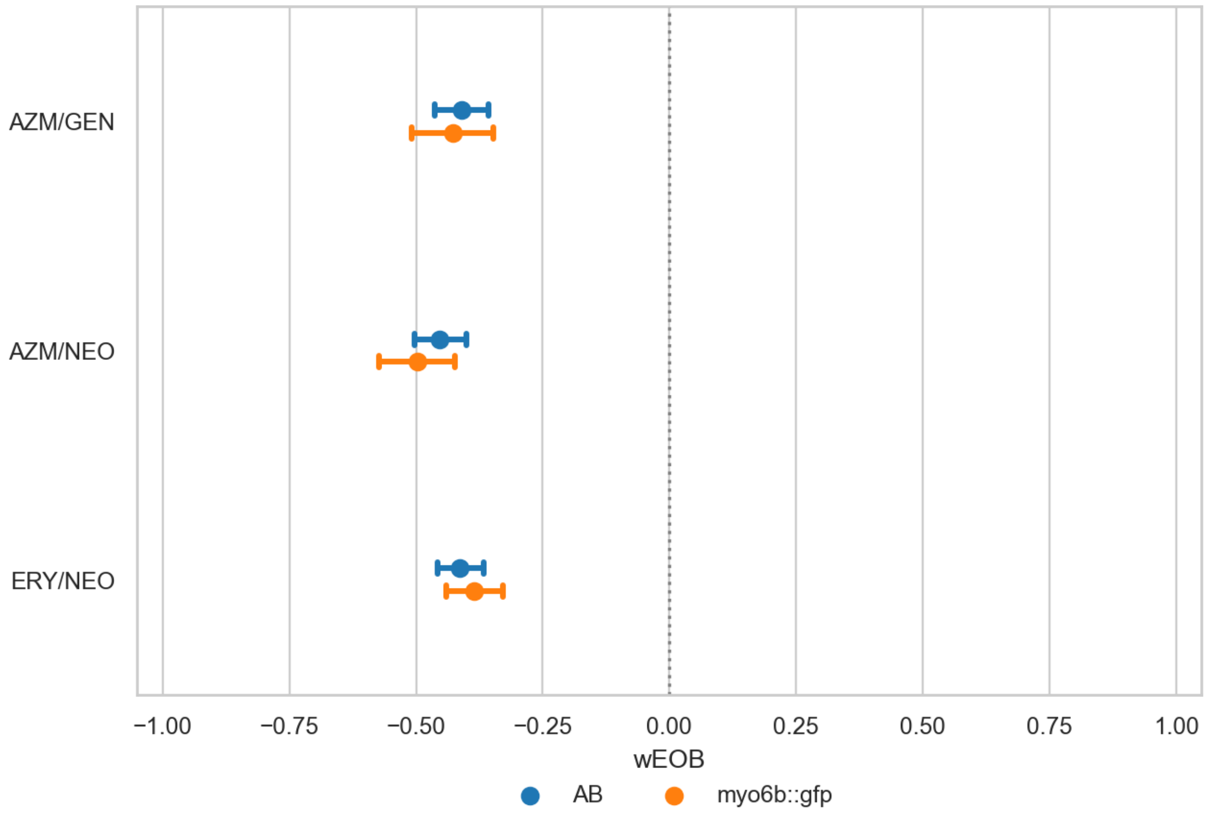

Figure S4. Comparison of aggregate wEOB value between strains. The aggregate wEOB for each combination tested in both strains shows strong antagonism and does not significantly differ between strains (AZM/GEN, $p=0.75$; AZM/NEO, $p=0.35$; ERY/NEO, $p=0.44$).

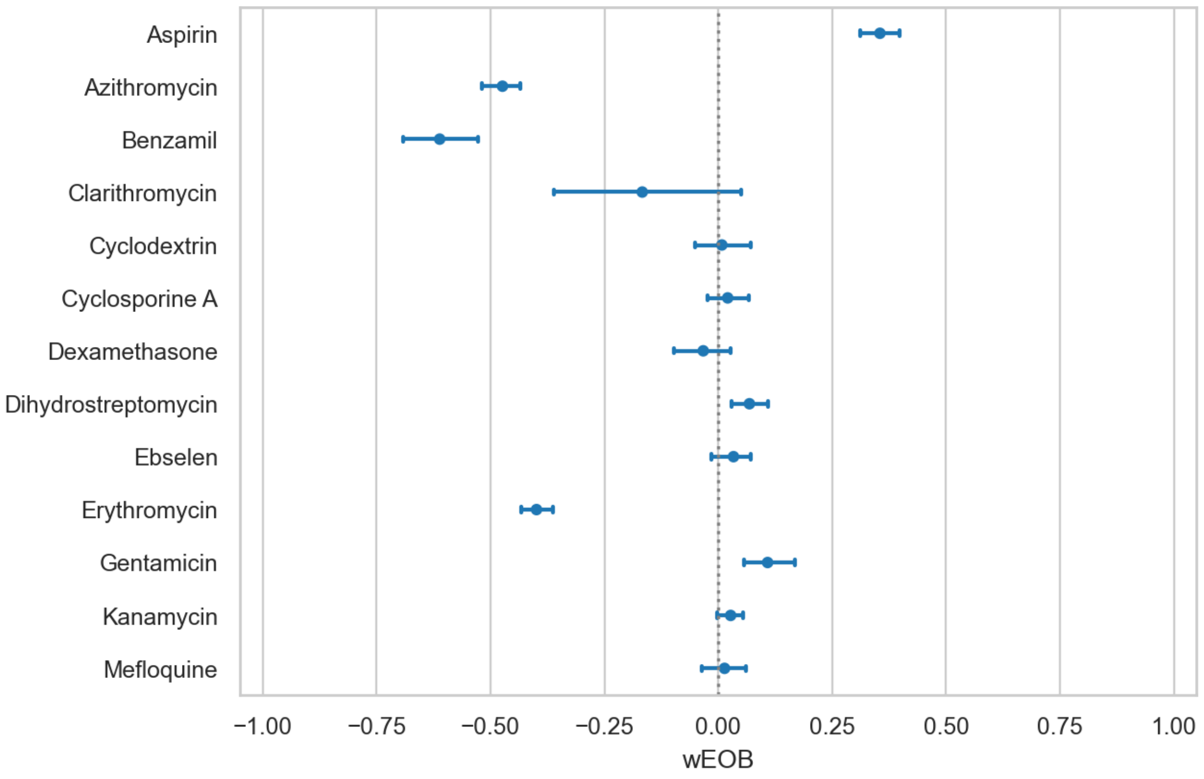

Figure S5. Overview of interaction scores observed between neomycin and various other drugs screened for significant interactions. Increasingly negative wEOB scores indicate increasing antagonism between NEO and the listed drug; increasingly positive wEOB scores indicate increasing synergy. Most drugs lie near zero, as would be expected for noninteractive compounds. The only listed compounds that deviate toward antagonism are the three macrolides tested, plus benzamil, a potent MET channel inhibitor well established in antagonizing NEO uptake.

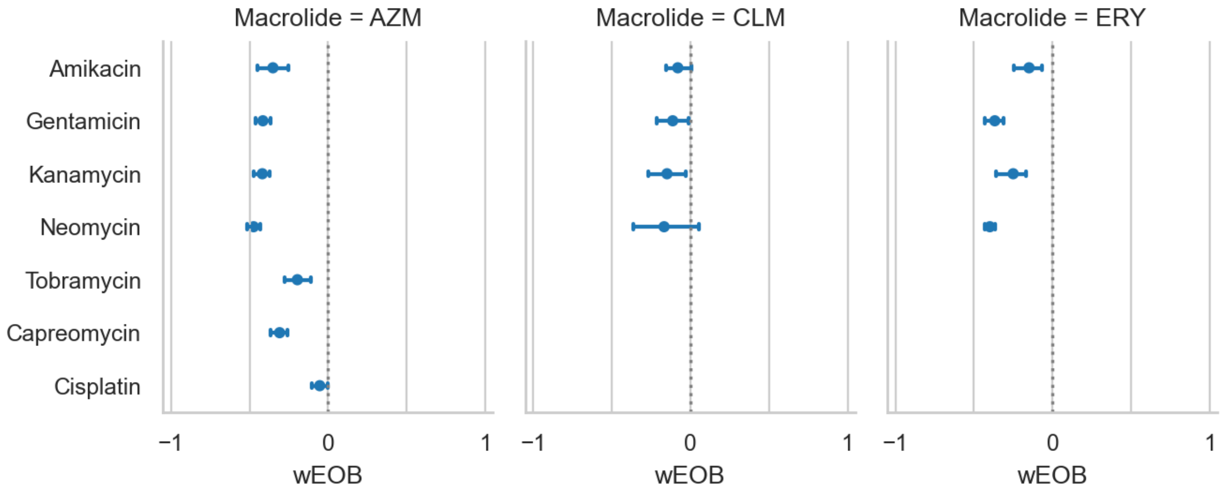

Figure S6. Overview of interaction scores between macrolide antibiotics and ototoxic drugs tested.

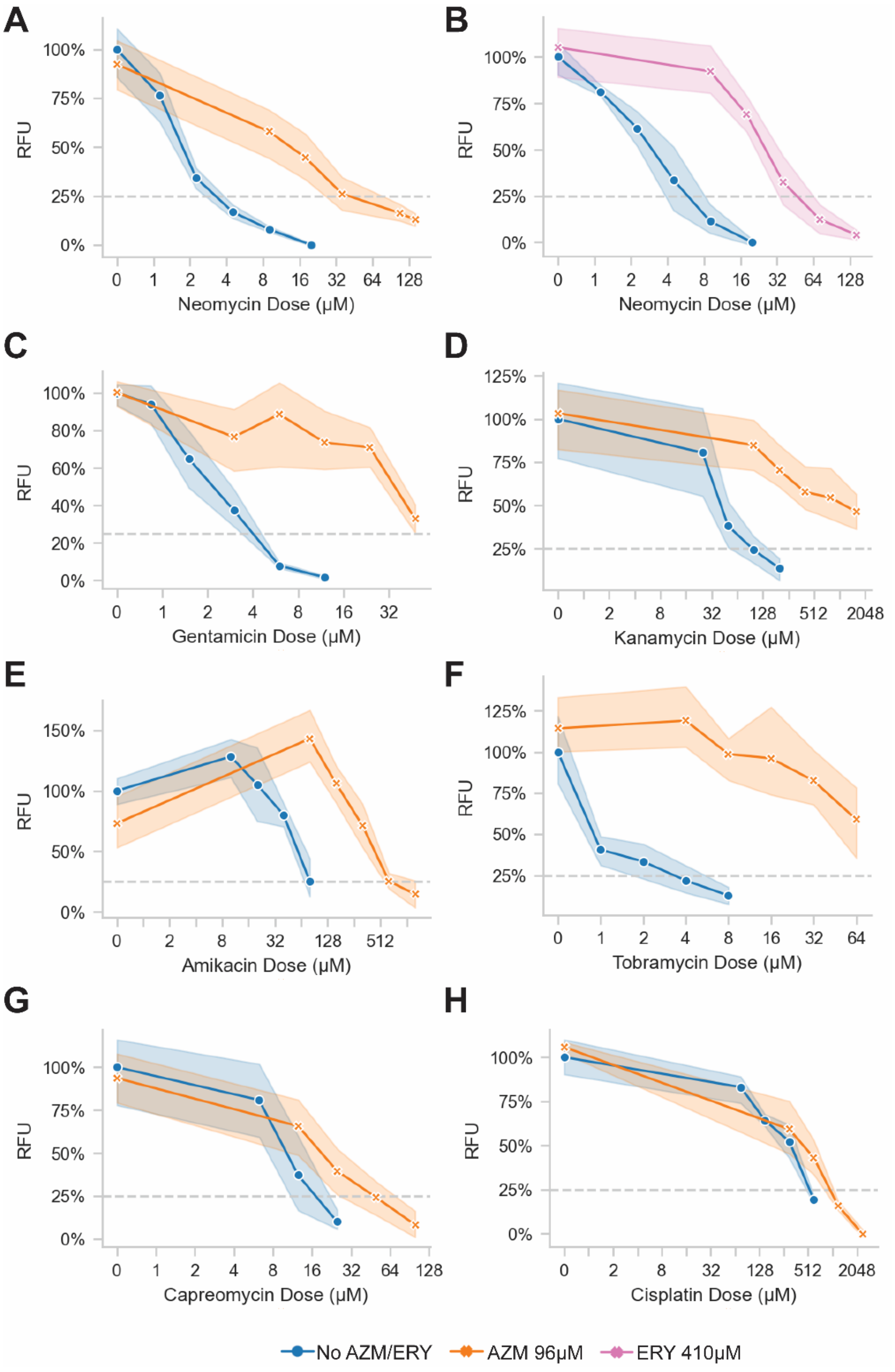

Figure S7. AZM antagonizes aminoglycoside-induced ototoxicity. We observe at least 8-fold increase in EC_{75} across all aminoglycoside combinations tested. Non-aminoglycoside ototoxins CAP and CIS are antagonized to a lesser extent. ERY antagonizes NEO-induced ototoxicity to a comparable extent to AZM.

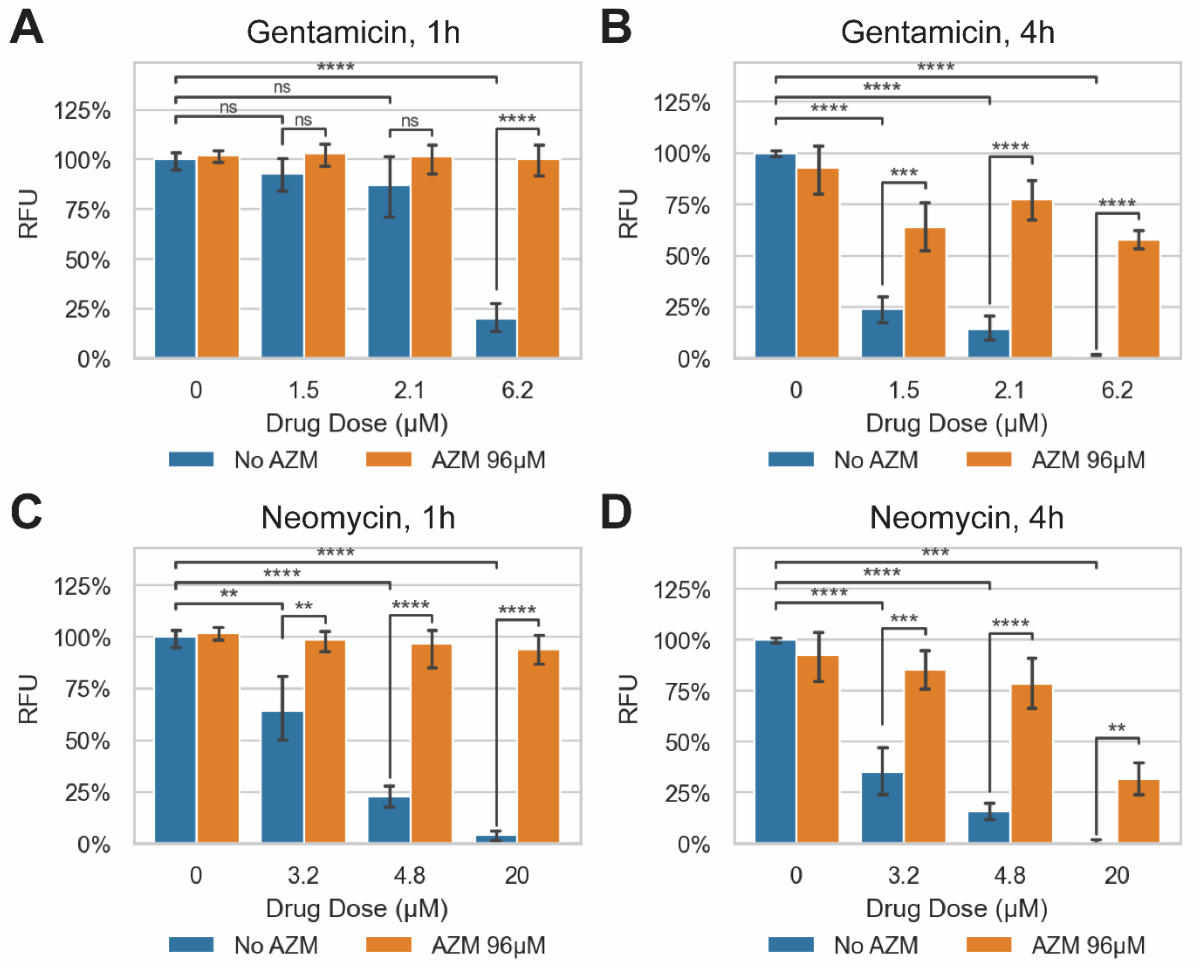

Figure S8. Extent of aminoglycoside-induced ototoxic damage and macrolide-conferred otoprotection as a function of time. Doses were chosen to represent estimated effective concentrations necessary to achieve 50%, 75% and 99% inhibition, respectively, as calculated from previous experiments at the 4-hour timepoint. AZM confers significant protection in all conditions where monotherapy shows significant damage as compared to untreated control. Corrected p-values: * = $p < 0.05$; ** = $p < 0.01$; *** = $p < 0.001$; **** = $p < 0.0001$.

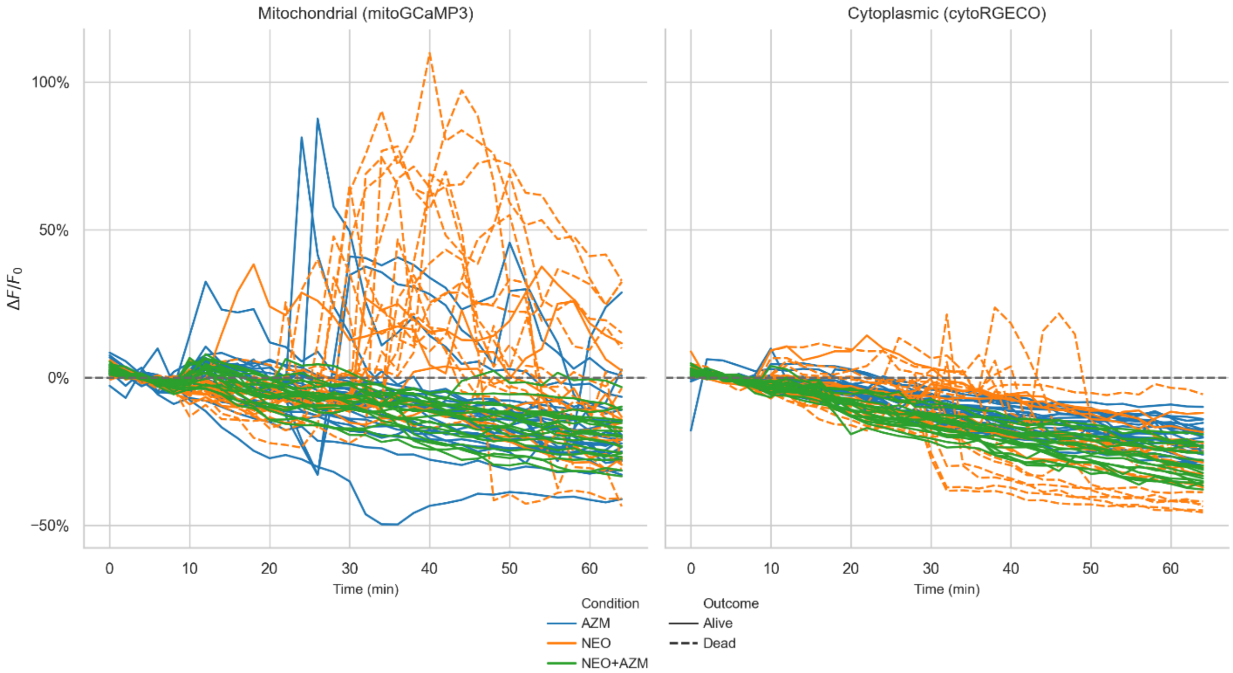

Figure S9. Quantification of mitochondrial (left) and cytoplasmic (right) Ca^{2+} levels in individual hair cells in response to AZM treatment (blue), NEO treatment (orange), or the two co-administered (green), as measured by *mitoGCaMP3* and *cytoRGECO* fluorescence signal. Drugs were administered at t = 10 min. A majority of cells exposed to NEO alone went on to die (14/21 cells from 9 neuromasts in 4 fish), while most exposed to AZM alone (24/25 cells from 11 neuromasts in 5 fish) and all treated with both drugs (19/19 cells from 10 neuromasts in 5 fish) survived.

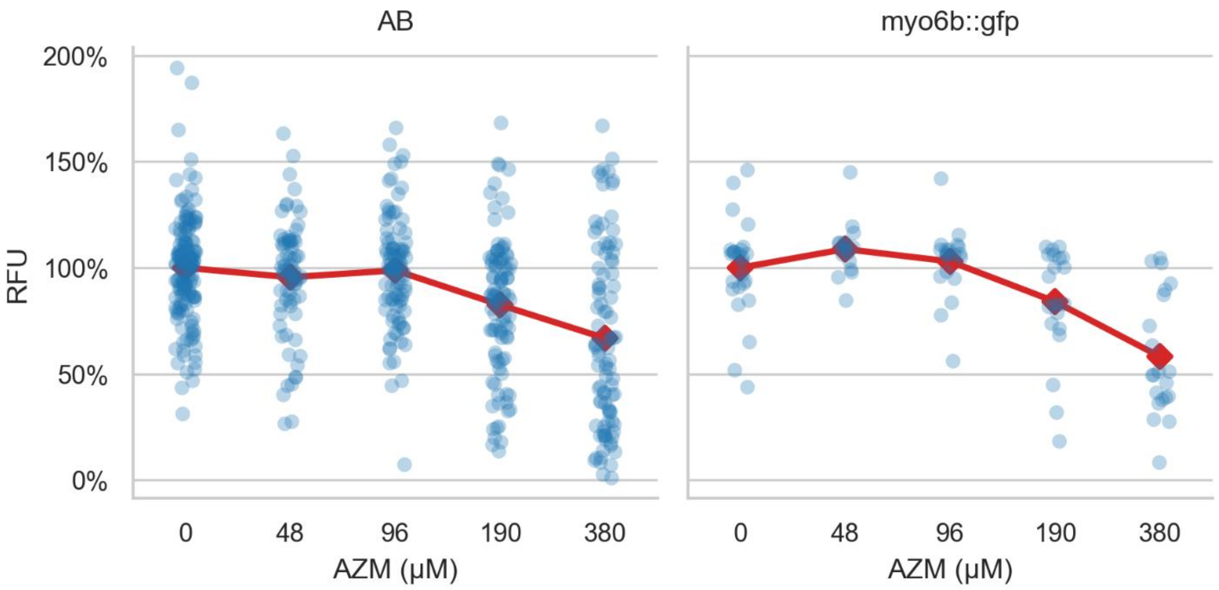

Figure S10. Hair cell damage dose response of azithromycin individual drug treatment. Aggregate dose-response curve when exposed to increasing doses of AZM in AB (A) and myo6b::gfp (B) fish. Significant hair cell damage is observed at high doses (Approximately 65% RFU remaining on average in 380µM AZM ($p < 1 \times 10^{-10}$)).

2. Supplementary Table

Uptake Inhibitor	*Name*	*Details*
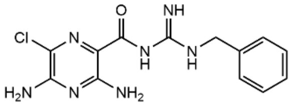	*Benzamil*	*MET channel inhibitor (PMID 22967486)*
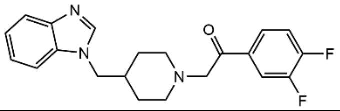	*UoS-7692*	*MET channel inhibitor (PMID 33735112)*
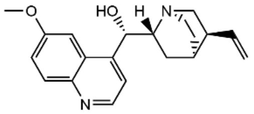	*Quinine*	*MET channel inhibitor (PMID 15181168)*
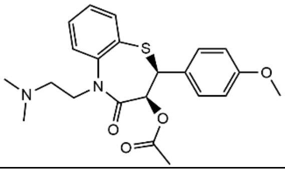	*Diltiazem*	*MET channel inhibitor (PMID 15181168)*
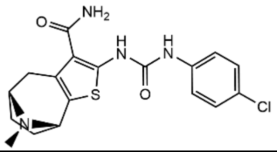	*ORC-13661*	*MET channel inhibitor (PMID 31391343)*
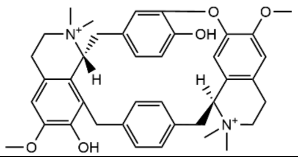	*Curare*	*MET channel inhibitor (PMID 15181168)*
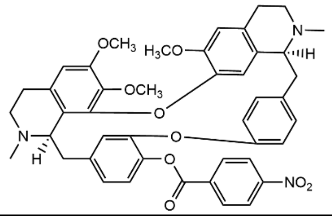	*E6 Berbamine*	*MET channel inhibitor (PMID 27065807)*
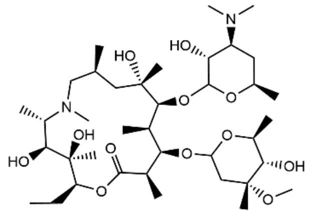	*Azithromycin*	*Prevents neomycin uptake by means other than MET channel inhibition (this study)*
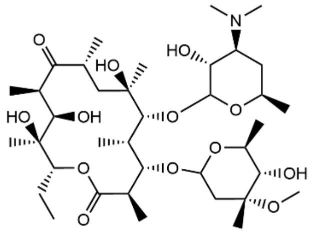	*Erythromycin*	*Prevents neomycin uptake by means other than MET channel inhibition (this study)*

Table S1. Comparison between macrolides and a range of known MET channel blockers. Molecules were visualized using ChemDraw 22.2.
